# Automatic opportunistic osteoporosis screening using low-dose chest computed tomography scans obtained for lung cancer screening

**DOI:** 10.1007/s00330-020-06679-y

**Published:** 2020-02-19

**Authors:** Yaling Pan, Dejun Shi, Hanqi Wang, Tongtong Chen, Deqi Cui, Xiaoguang Cheng, Yong Lu

**Affiliations:** 1 Department of Radiology, Ruijin Hospital, Shanghai Jiao Tong University School of Medicine, Shanghai, 200025 China; 2 LinkingMed, Beijing, 100000 China; 3 Department of Radiology, Beijing Jishuitan Hospital, Beijing, 100035 China

**Keywords:** Bone mineral density, Deep learning, Osteoporosis, Screening

## Abstract

**Objective:**

Osteoporosis is a prevalent and treatable condition, but it remains underdiagnosed. In this study, a deep learning-based system was developed to automatically measure bone mineral density (BMD) for opportunistic osteoporosis screening using low-dose chest computed tomography (LDCT) scans obtained for lung cancer screening.

**Methods:**

First, a deep learning model was trained and tested with 200 annotated LDCT scans to segment and label all vertebral bodies (VBs). Then, the mean CT numbers of the trabecular area of target VBs were obtained based on the segmentation mask through geometric operations. Finally, a linear function was built to map the trabecular CT numbers of target VBs to their BMDs collected from approved software used for osteoporosis diagnosis. The diagnostic performance of the developed system was evaluated using an independent dataset of 374 LDCT scans with standard BMDs and osteoporosis diagnosis.

**Results:**

Our deep learning model achieved a mean Dice coefficient of 86.6% for VB segmentation and 97.5% accuracy for VB labeling. Line regression and Bland-Altman analyses showed good agreement between the predicted BMD and the ground truth, with correlation coefficients of 0.964–0.968 and mean errors of 2.2–4.0 mg/cm^3^. The area under the curve (AUC) was 0.927 for detecting osteoporosis and 0.942 for distinguishing low BMD.

**Conclusion:**

The proposed deep learning-based system demonstrated the potential to automatically perform opportunistic osteoporosis screening using LDCT scans obtained for lung cancer screening.

**Key Points:**

*• Osteoporosis is a prevalent but underdiagnosed condition that can increase the risk of fracture.*

*• A deep learning-based system was developed to fully automate bone mineral density measurement in low-dose chest computed tomography scans.*

*• The developed system achieved high accuracy for automatic opportunistic osteoporosis screening using low-dose chest computed tomography scans obtained for lung cancer screening.*

## Introduction

Osteoporosis is a prevalent and latent metabolic bone disease characterized by loss of bone mass and consequent susceptibility to fracture. With the progressively aging population, the number of patients in China with osteoporosis or osteoporotic fracture is projected to reach approximately 212 million and 5.99 million, respectively, by 2050 [1, 2], which will cause substantial economic costs, morbidity, and mortality. Currently, osteoporosis remains substantially underdiagnosed. More than half of patients with osteoporotic fracture have never undergone osteoporosis screening [3]. Therefore, early screening and monitoring of osteoporosis are crucial for timely prevention and treatment of osteoporotic fracture.

Bone mineral density (BMD), directly related to bone strength, is widely used to diagnose and monitor osteoporosis in clinical practice [4]. Quantitative computed tomography (QCT) is increasingly used to measure vertebral BMD from clinical computed tomography (CT) scans and has higher sensitivity than dual-energy X-ray absorptiometry (DXA) for diagnosing osteoporosis and predicting the risk of osteoporotic fracture [5]. Compared with DXA, QCT is less susceptible to confounding factors such as spinal degenerative changes, aortic calcification, bone size, and body mass index, and can selectively measure trabecular BMD [6]. Trabecular BMD is considered a more sensitive marker for changes in overall bone strength because it is generally lost more rapidly than cortical BMD when the disease progresses [7].

Low-dose chest computed tomography (LDCT) is popularly used for early lung cancer screening with less ionizing radiation and has been demonstrated to significantly reduce mortality from lung cancer [8]. LDCT and QCT may be an attractive combination to screen for both lung cancer and osteoporosis with a single LDCT scan to limit radiation dose and expense. Four factors make the combination plausible: both lung cancer and osteoporosis tend to affect the population aged 50 years or older; annual lung cancer screening using LDCT is widely implemented; LDCT scans generally cover the upper lumber vertebrae [9]; and asynchronous QCT has been introduced in clinical workflow for convenient and accurate BMD measurement of the spine [10]. Recently, CT covering part of the spine has been suggested to detect patients with osteoporosis [11, 12]. The utility of vertebral CT numbers derived from LDCT for detecting osteoporosis has been confirmed [9].

However, QCT image analysis still requires frequent manual operations including localization of vertebral bodies (VBs) and placement of volumes of interest (VOIs), which imposes heavy and reduplicative tasks in large-scale osteoporosis screening. Recently, deep learning (DL), especially convolutional neural network (CNN), has dramatically improved the performance of vertebrae recognition and segmentation [13, 14]. DL is expected to eliminate the manual operation in BMD measurement and thus liberate radiologists for more meaningful tasks while also reducing the cost of screening. Previous studies focused on automatic BMD assessment from abdominopelvic and spinal CT scans [15, 16]. In this study, we propose harnessing DL to develop a system, as an alternative to QCT image analysis software, which will automatically measure BMD and detect osteoporosis from LDCT scans during lung cancer screening.

## Materials and methods

This study was approved by the local ethical review board (IRB No. 201875) and informed consent was waived for a retrospective analysis.

### Subjects

From the electronic database of our hospital, we retrieved the data of individuals who underwent paired LDCT and QCT examinations for screening both lung cancer and osteoporosis from April 1 to October 31, 2018. All individuals were scanned from the apical lung to the lower edge of L2 on iCT 256 scanners (Phillips Medical Systems) that were calibrated daily to ensure accurate CT numbers. Scan parameters were as follows: 120 kVp, average 30 mAs, 5-mm section collimation, 500-mm scan field of view, and 1-mm standard reconstruction interval. To calibrate the linear function between CT numbers and BMD values, quality assurance (QA) phantom data were acquired once a month through separately scanning an asynchronous phantom (Mindways Software, Inc.) with the same scan parameters. The BMD of all individuals was measured on three consecutive VBs: T12 to L2 using QCT image analysis software (QCT Pro 6.1 version, Mindways Software, Inc.). The 9-mm-high VOIs capturing the trabecular bone were manually placed in the center of the target VBs, avoiding the basivertebral veins, cortical bone, and any focal pathology. In this study, we excluded individuals who had a history of prior spinal surgery, primary or metastatic tumors, or fractured vertebrae. Finally, 574 individuals were enrolled in this study. Two hundred LDCT scans were manually annotated by an experienced radiologist for the contours and the anatomical names of all VBs and were used to develop the deep learning-based system. The remaining 374 unannotated LDCT scans of 196 men and 178 women (mean age 62.6 ± 7.6 years, range 50–88 years) were used to evaluate the developed system.

### Development of the BMD measurement system

**Fig. 1:**
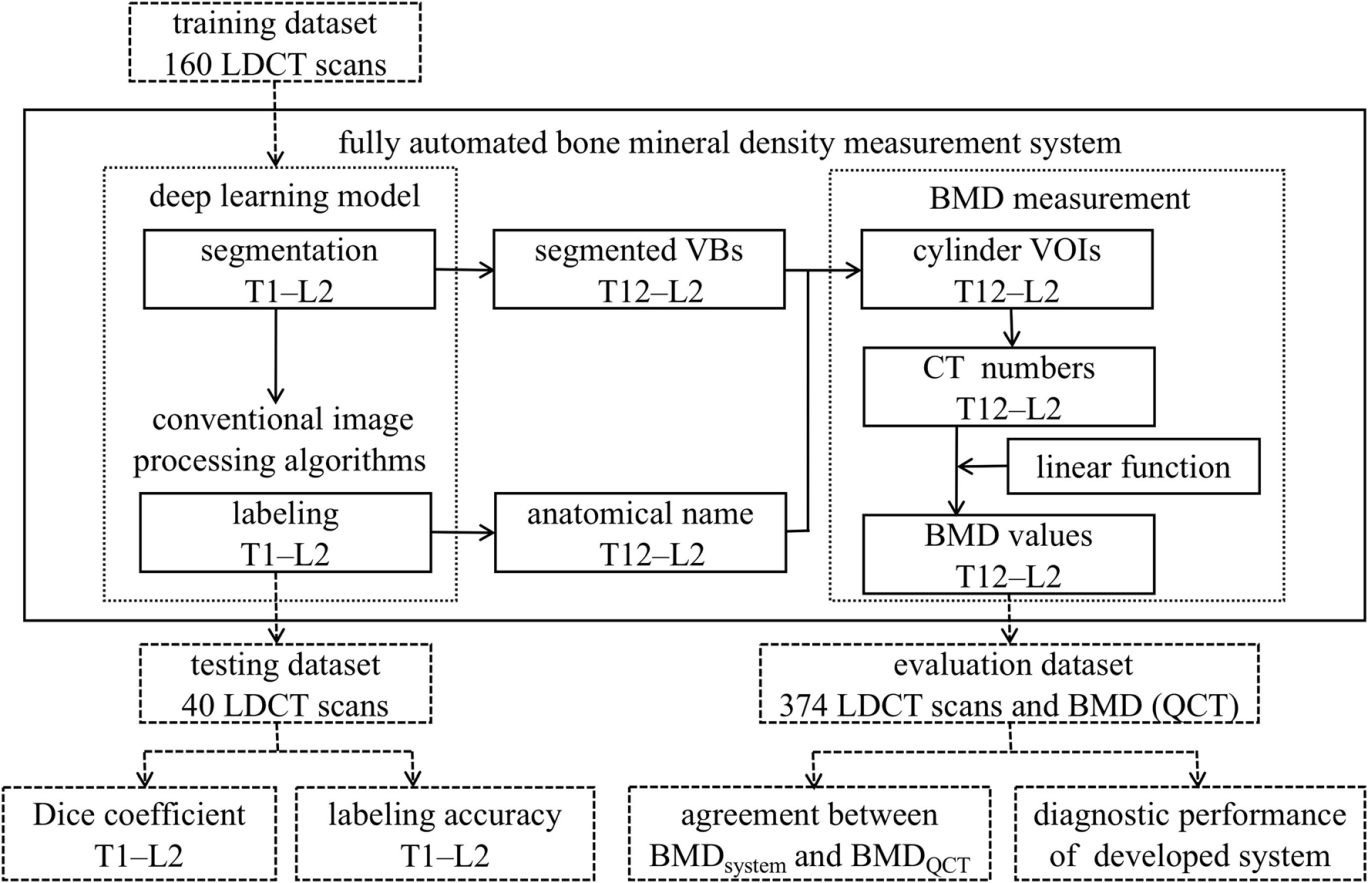
Flow chart illustrating the development and evaluation of the fully automated bone mineral density measurement system. BMD_system_, the BMD measured by the developed system; BMD_QCT_, the BMD measured by QCT image analysis software

The development of a fully automated BMD measurement system consisted of three main stages (Fig. 1). First, an end-to-end DL model was trained to segment VBs arbitrarily divided into three categories: T1–T6, T7–T12, and L1–L2. The anatomical names of segmented VBs were deduced using conventional image processing algorithms. Second, trabecular areas of the target VBs (T12, L1, L2) were extracted based on the segmentation mask through geometric operations. Finally, mean CT numbers within the cylinder VOIs of the target VBs were mapped to their BMD values using a linear function, which was used to predict BMD during inference.

#### VB segmentation

**Fig. 2:**
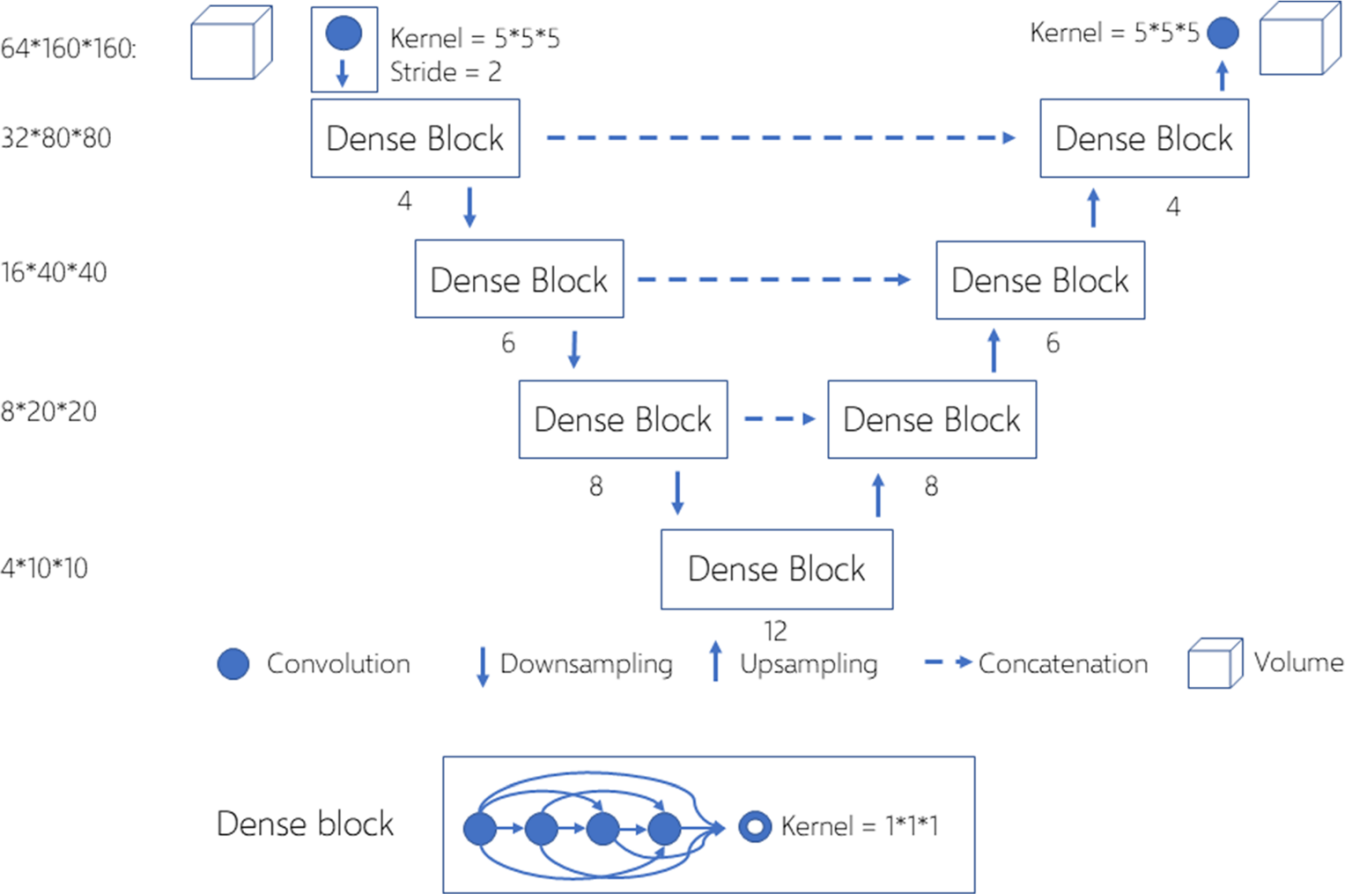
3D U-net with dense blocks. The default kernel size for 3D convolution was 3 × 3 × 3, the growth rate of the dense block was 8, and the number of layers in the dense blocks varied for different levels indicated by an integer below the block. For each dense block, the final output went through a 1 × 1 × 1 3D convolution to reduce dimensions

We framed the segmentation task as a four-class voxel-level classification problem, one class for background and the other three classes for three categories of VBs, namely T1–T6, T7–T12, and L1–L2. To address this task, we developed a 3D CNN model with U-net architecture [17] and dense connections [18] (Fig. 2) that receives 3D patches sampled from individual LDCT scans as input and outputs the same-sized four-class segmentation mask. U-net is a classical CNN model for segmentation in medical imaging and has a symmetric decoder-encoder architecture and skip connections to combine low- and high-level features. From 2D to 3D, the parameters and computation of a U-net increase exponentially, so we added dense connections between the plain convolutional networks within each level to maintain a good balance between performance and computation. Dense connections could reduce the number of parameters through feature reuse to improve the overall efficiency of the CNN and has been successfully used in segmenting multiple organs [19].

#### VB labeling

**Fig. 3:**
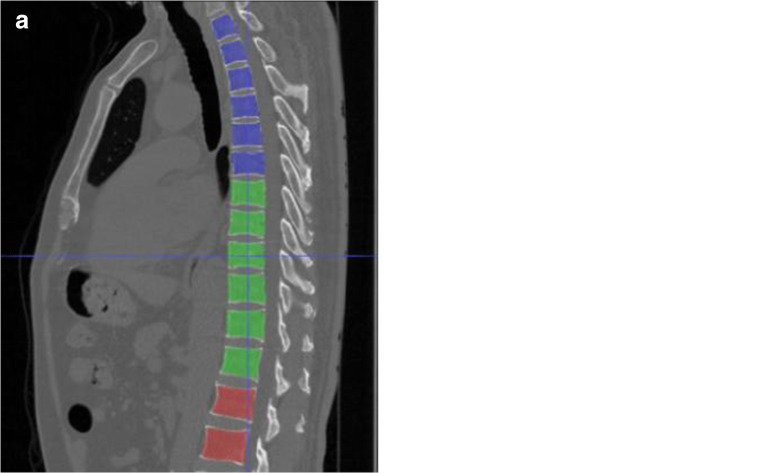
**a** VB masks with three categories were predicted by the DL model and visualized by ITK-SNAP. **b** Each VB mask was renamed as its own anatomical name using conventional image processing algorithms and represented by distinct colors. Class one: T1–T6 (blue); class two: T7–T12 (green); class three: L1–L2 (red)

Following the segmentation, we utilized conventional image processing algorithms to isolate and label each individual VB. The slice thickness of LDCT scans was 1 mm, which ensured that the VBs in LDCT scans were not in contact and their corresponding regions in a binary mask could be separated by image labeling algorithms [20]. We first aggregate the prediction results of all 3D input patches into one mask volume (Fig. 3a), and then apply post processing to give anatomical names to individual VBs and thus realize VB labeling (Fig. 3b). VB masks were visualized by ITK-SNAP [21]. In the post processing, we used image labeling algorithms to label class three VBs as distinct lumbar VBs starting from L1 top down and to label class one and class two as distinct thoracic VBs from T12 bottom up.

#### Training and testing

We trained and tested the 3D dense connected U-net using Keras with tensorflow as the backend on two Nvidia GTX 1080 Ti GPUs (16 memory). Due to the computation and memory constraint, the input size of our 3D model was set to 64 × 160 × 160. To include all VBs in the input patches of this size, we first rescaled all axial slices using bilinear interpolation to 2 mm × 2 mm and then cropped the slices to 160 × 160 based on the body center found by thresholding. One 3D input patch contains at most four VBs. We randomly split 200 annotated LDCT scans into two datasets (160 for training and 40 for testing). In total, we sampled over 7000 3D patches in a window sliding fashion and augmented each input patch with translation and rotation in the axial plane on the fly during training. The loss function was the Dice loss. Adam was used as our optimizer with the initial learning rate of 1e-4 which was reduced by half when the Dice metrics did not decrease for three consecutive epochs. It took 48 h to train the 3D dense-connected U-net for 300 epochs. Note that the DL model was trained from scratch and tested by taking manual segmentation and labeling as ground truth.

#### BMD measurement

**Fig. 4:**
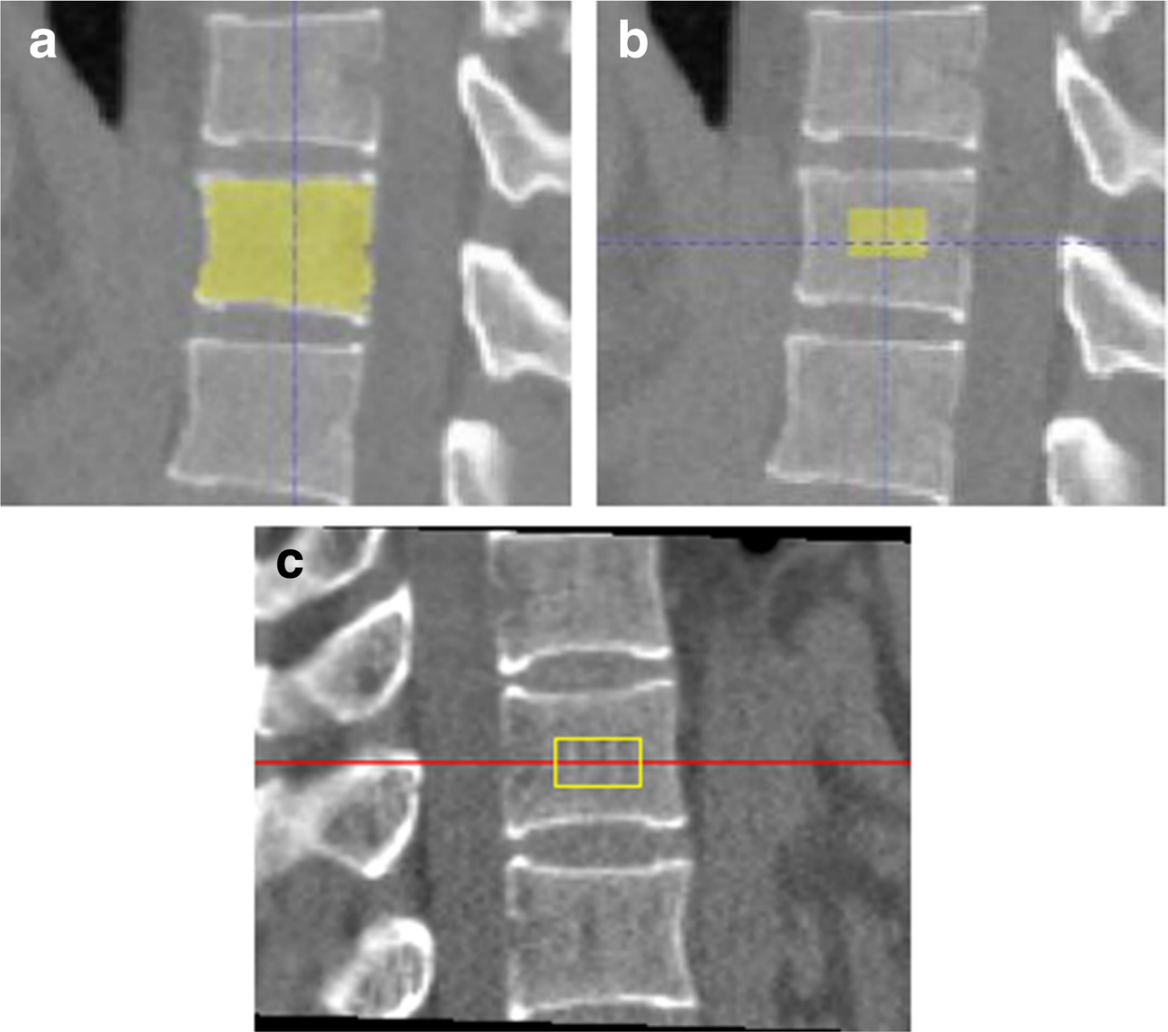
The developed system and QCT image analysis software in creating VOIs for BMD measurement. **a** Segmented VB generated by the DL model. **b** The automatic VOI generated by the developed system. **c** The semi-automatic VOI generated by the QCT image analysis software as reference

With the segmentation masks of the target VBs, we automatically extracted the trabecular area in accordance with the cylinder VOIs of QCT image analysis software through geometric operations, as illustrated in Fig. 4. As the cylinder VOI was 9 mm high and the slice thickness of the LDCT was 1 mm, we transformed the task as extracting an axial ellipse of the same location and size from the middle nine slices of a target VB mask. Both location and size of the axial ellipse was based on the 2D VB region of the middle slice of the target mask. The location of the ellipse was centered within the VB region; the size was determined by arbitrarily setting the area of the ellipse to 30% of the VB area and the short arm to 20% of the maximum row length of the VB region, with the long arm inferred. These percentage parameters were based on experiments and the fact that cortical thickness of VBs ranges from 0.45 to 1.02 mm [22] to ensure successful extraction of the trabecular area. Then, the mean CT numbers within the cylinder VOIs at each vertebral level (T12 to L2) were calculated and mapped to BMD values (mg/cm^3^) measured by QCT using one-degree linear function. The linear model was fitted with the paired mean CT numbers and BMD data of the target VBs in the 160 cases of training data. Note that the one-degree linear function in the developed system was allowed to be adjusted for future input of calibrated function obtained from QA phantom scanning. According to the standard clinically used L1–L2 BMD average, bone mass was defined as normal (> 120 mg/cm^3^), osteopenia (80–120 mg/cm^3^), or osteoporosis (< 80 mg/cm^3^) [6]. The BMD values of T12 to L2 and bone mass assessment are the final output of the developed system.

### Statistical analysis

SPSS 22.0 software (IBM SPSS Statistics for Windows) was used for statistical analysis. Segmentation performance was evaluated using the Dice coefficient that can effectively quantify the spatial overlap between segmentation and ground truth [13]. The percentage of VBs that were assigned the correct anatomical name was presented as labeling accuracy. All continuous variables were tested for normality before analysis. Linear regression and Bland-Altman analyses were performed to compare the BMD measurements predicted by the developed system with the ground truth generated by QCT. Using QCT as a reference standard, the area under the curve (AUC) from receiver operating characteristic (ROC) analysis and the sensitivity, specificity, and positive and negative predictive values were calculated to evaluate the diagnostic performance of the developed system for osteoporosis and low BMD (osteoporosis or osteopenia), respectively. All analyses were two-tailed and *p* < 0.05 was considered significant.

## Results

### VB segmentation and labeling

The proposed method achieved a mean Dice coefficient of 86.6% and labeling accuracy of 97.5% for VB segmentation and labeling, respectively, on the testing dataset (Table 1). There was a general trend of increasing Dice coefficients from upper VBs (T1–T10) to lower VBs (T11–L2). Only one LDCT scan presented an error in the VB labeling, where seven VBs were predicted as class two (T7–T12) VBs resulting in 13 thoracic VBs, and VB L1 was consequently mislabeled as a thoracic VB.

**Table 1:** Segmentation and labeling results of VBs calculated from LDCT scans

VBs	T1	T2	T3	T4	T5	T6	T7	T8	T9	T10	T11	T12	L1	L2	Overall
Mean Dice coefficient (%)															
	81.4	84.1	83.7	85.0	86.5	86.9	87.6	87.9	86.9	87.6	88.2	88.2	88.9	89.0	86.6
Labeling accuracy (%)															
	97.5	97.5	97.5	97.5	97.5	97.5	97.5	97.5	97.5	97.5	97.5	97.5	97.5	97.5	97.5

### Agreement between the developed system and QCT for BMD measurement

**Fig. 5:**
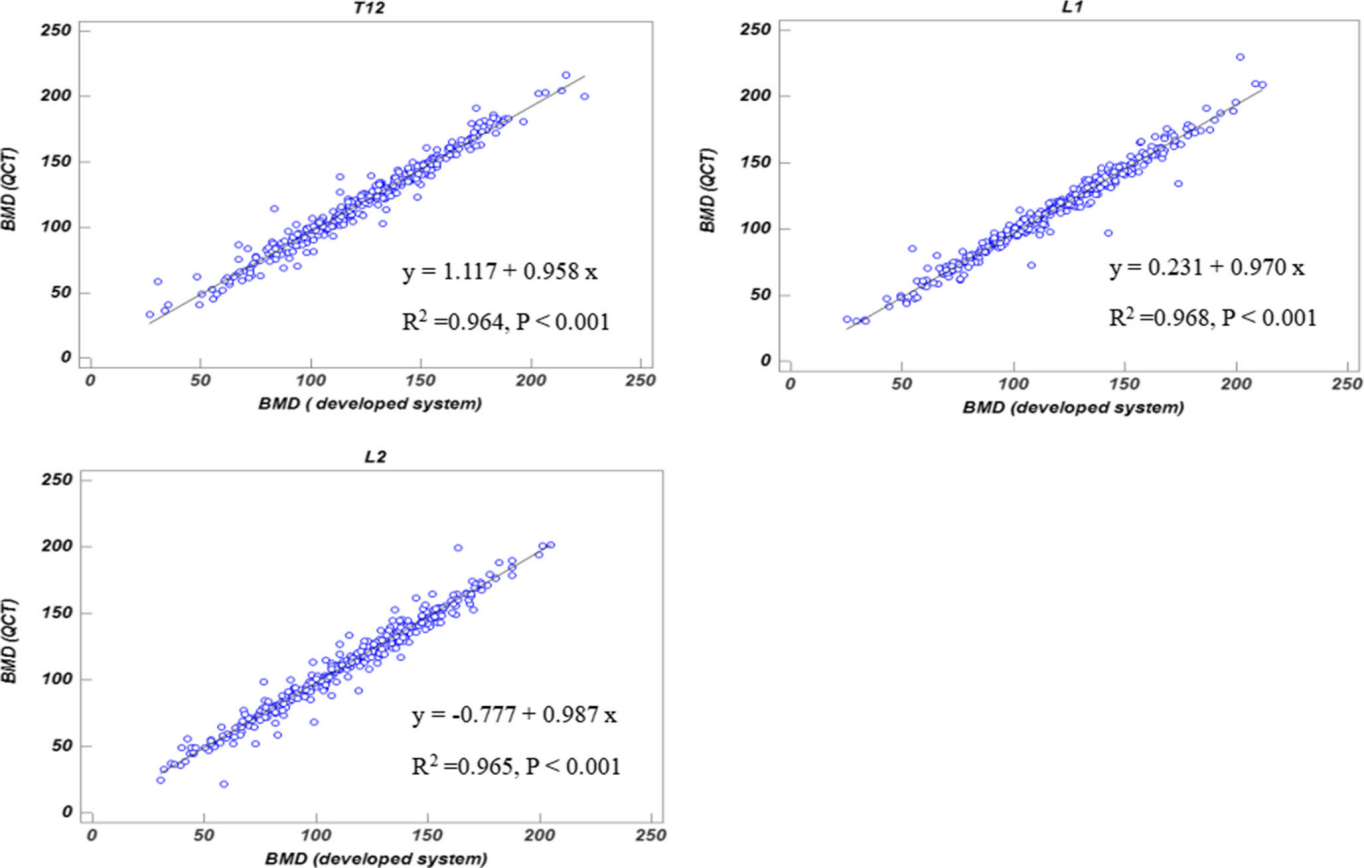
Linear regressions of BMD values between the developed system and QCT at each vertebral level from T12 to L2

**Fig. 6:**
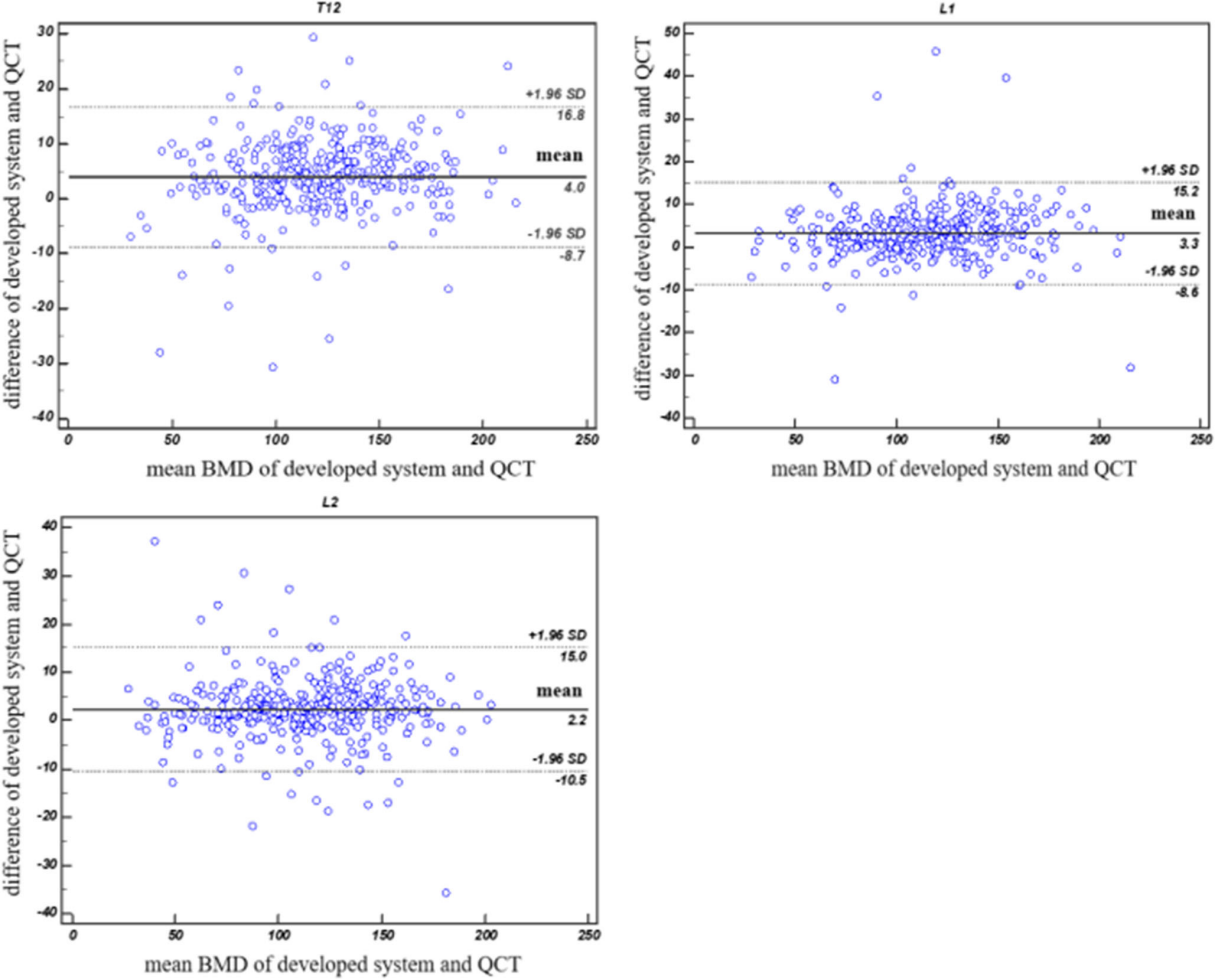
A Bland-Altman plot comparing BMD values obtained using the developed system and QCT at each vertebral level from T12 to L2. The mean difference (solid line) and limits of agreement (dotted line) are shown

With the evaluation data, linear regression analysis demonstrated an excellent correlation between the developed system and QCT for BMD measurement of the three target VBs, with a correlation coefficient (*R*^2^) of 0.964–0.968 (Fig. 5). Furthermore, the slopes of linear regressions with the developed system and QCT were near one, indicating that the developed system could accurately measure vertebral BMD. Bland-Altman analysis also revealed good agreement between the developed system and QCT for BMD measurement at each vertebral level in Fig. 6. Compared with the results of QCT, BMD measurement by the developed system generated mean errors of 2.2–4.0 mg/cm^3^. The 95% limits of agreement from T12 to L2 were (− 8.7, 16.8) mg/cm^3^, (− 8.6, 15.2) mg/cm^3^, and (− 10.5, 15.0) mg/cm^3^, respectively.

### Diagnostic performance of the developed system for osteoporosis and low BMD

The diagnostic performance of the developed system for osteoporosis and low BMD is shown in Table 2. The AUC of the developed system for detecting osteoporosis was 0.927, which was lower than the AUC (0.942) for distinguishing low BMD from normal bone mass. For detection of osteoporosis, the developed system achieved 90.70% sensitivity and 99.26% specificity among women, and 75% sensitivity and 100% specificity among men. Five men with osteoporosis (BMD range 72.71 to 79.97 mg/cm^3^) were misdiagnosed with osteopenia because the developed system slightly overestimated their BMD. Among total individuals, the sensitivity and specificity of the developed system for detecting osteoporosis and distinguishing low BMD from normal bone mass were 85.71% and 99.68% and 90.37% and 98.08%, respectively.

**Table 2:** The diagnostic performance of the developed system for detecting osteoporosis and distinguishing low BMD from normal bone mass, using QCT as the reference standard

Diagnosis	AUC (95% CI)	Sensitivity (n/N)	Specificity (n/N)	PPV (n/N)	NPV (n/N)
Women (*n* = 178)					
Osteoporosis	0.950 (0.907–0.977)	90.70% (39/43)	99.26% (134/135)	97.50% (39/40)	97.10% (134/138)
Low BMD	0.933 (0.885–0.965)	90.00% (108/120)	96.55% (56/58)	98.18% (108/110)	82.35% (56/68)
Men (*n* = 196)					
Osteoporosis	0.875 (0.820–0.918)	75.00% (15/20)	100.00% (176/176)	100.00% (15/15)	97.24% (176/181)
Low BMD	0.949 (0.908–0.975)	90.82% (89/98)	98.98% (97/98)	98.89% (89/90)	91.51% (97/106)
Total (*n* = 374)					
Osteoporosis	0.927 (0.896–0.951)	85.71% (54/63)	99.68% (310/311)	98.18% (54/55)	97.18% (310/319)
Low BMD	0.942 (0.914–0.964)	90.37% (197/218)	98.08% (153/156)	98.50% (197/200)	87.93% (153/174)

## Discussion

This study was motivated by recent advances of DL in medical imaging [19] and aimed to fully automate BMD measurement and osteoporosis detection in opportunistic osteoporosis screening using LDCT scans obtained for lung cancer screening. To our knowledge, this was the first study to automate BMD measurement using DL and evaluate the performance against QCT in LDCT scans.

VB segmentation and labeling in volumetric LDCT scans are a prerequisite of BMD measurement. These tasks are challenging due to the similar structures of adjacent VBs, the spatial interrelation between VBs and the surrounding bone anatomies, such as ribs, and pathologies including degenerative changes of vertebrae and vertebral disks [23, 24]. Using only a small training dataset (160 LDCT scans), our method achieved a mean Dice coefficient of 86.6% and labeling accuracy of 97.5% for VB segmentation and labeling. In terms of the absolute metric, our method performed worse in VB segmentation than that used in a previous study [24–26] but yet surpassed previously reported results [13, 27] in VB labeling. Nevertheless, since different datasets were used in assessment of different methods, direct comparison of different methods should be interpreted cautiously. The performance of our method ultimately is determined by the 3D patch CNN segmentation model because VB labeling depended on the results of VB segmentation. The suboptimal performance of our segmentation model might result from several factors. First, the overall training dataset was small. Second, the annotation, provided by one radiologist, might be subjected to intra-observer variability. Third, the rescaling of the axial plane by a factor of two inflicted information loss and lowered the ability of our model to optimally segment the edges of VBs.

We used QCT instead of DXA of the lumbar spine as a reference standard for BMD measurement because QCT could provide more reliable evaluation of the performance of our developed system. Although DXA of the lumbar spine is the most commonly used reference standard for the diagnosis of osteoporosis [28] and is adopted by most studies to evaluate the diagnostic performance of CT numbers for detecting osteoporosis or low BMD [9, 12, 29], the BMD of DXA is not derived from CT numbers and it is difficult to find any individuals who underwent LDCT and DXA within a short interval. The BMD values measured by our developed system have been demonstrated to have a strong statistically significant correlation and to be in good agreement with values obtained by QCT. These results suggest that vertebral BMD measurement can be automatically obtained by the developed system with good accuracy. The BMD measurement by the developed system at lumber vertebral levels (L1 to L2) achieved slightly better correlation and agreement with QCT compared with BMD measurement at thoracic vertebral level (T12). Following the ISCD’s recommendations that L1–L2 vertebrae should be scanned for patients undergoing 3D QCT examination [30], the average L1–L2 BMD is the standard reportable metric in the developed system. Moreover, additional experimental BMD of T12 also was presented as the reference value when L1 or L2 vertebrae presented lesions such as fracture, tumor, sclerosis, or cysts. Compared with the user-supported systems [23], our developed system can achieve fully automated VB segmentation and labeling as well as BMD measurement without manual operation. The outputs of the developed system could be incorporated into a standard LDCT scan interpretation interface, only requiring checks from radiologists.

With the increasing use of LDCT for lung cancer screening, BMD measurement in LDCT scans could be an economical and safe alternative strategy to screen individuals at high risk of osteopenia and osteoporosis. Our developed system achieved 85.71% sensitivity and 99.68% specificity for diagnosing osteoporosis, and 90.37% sensitivity and 98.08% specificity for distinguishing low BMD from normal bone mass in LDCT scans. Thus, the developed system has the potential to be applied for opportunistic osteoporosis or low BMD screening using LDCT scans obtained for lung cancer screening, decreasing the number of overlooked or undiagnosed cases of osteoporosis or low BMD.

There were a few limitations to this study. First, the retrospective inclusion of individuals who underwent paired LDCT and QCT examinations may have caused selection bias. Second, all LDCT scans were obtained on scanners produced by a single manufacturer in this study. Manufacturer-related differences in acquisition or reconstruction settings might affect VB segmentation and labeling. Further confirmation in LDCT scans using scanners from other manufacturers and institutions will be required to prove the consistency, robustness, and transferability of this system. Third, the segmentation model did not make the most use of the annotation information and the labeling method would inherit the error produced by the segmentation model. Future studies may build an independent labeling method to calibrate the result of segmentation.

## Conclusion

For fully automated osteoporosis detection in LDCT scans obtained for annual lung cancer screening, a deep learning-based BMD measurement system was developed in this study. Using QCT as the reference standard, the performance of the developed system was evaluated. Strong correlation and good agreement were achieved at each vertebral level from T12 to L2 between the developed system and QCT for BMD measurement. The developed system automatically detected osteoporosis and low BMD with a high sensitivity and specificity in LDCT scans. Based on the reduced requirement for manual operation and the high accuracy, the developed system may be a promising tool to automatically measure vertebral BMD in opportunistic osteoporosis screening using LDCT scans obtained for lung cancer screening.

## Abbreviations

AUC: Area under the curve
BMD: Bone mineral density
CNN: Convolutional neural network
CT: Computed tomography
DL: Deep learning
DXA: Dual-energy X-ray absorptiometry
LDCT: Low-dose chest computed tomography
QA: Quality assurance
QCT: Quantitative computed tomography
VB: Vertebral body
VOI: Volume of interest
